# Large-scale differences in microbial biodiversity discovery between 16S amplicon and shotgun sequencing

**DOI:** 10.1038/s41598-017-06665-3

**Published:** 2017-07-31

**Authors:** Michael Tessler, Johannes S. Neumann, Ebrahim Afshinnekoo, Michael Pineda, Rebecca Hersch, Luiz Felipe M. Velho, Bianca T. Segovia, Fabio A. Lansac-Toha, Michael Lemke, Rob DeSalle, Christopher E. Mason, Mercer R. Brugler

**Affiliations:** 1 Sackler Institute for Comparative Genomics, American Museum of Natural History, New York, NY 10024 USA; 2 Richard Gilder Graduate School, American Museum of Natural History, New York, NY 10024 USA; 3 Stiftung Tierärztliche Hochschule Hannover, ITZ - Ecology and Evolution, Bünteweg 17d, D-30559 Hannover, Germany; 4 Department of Physiology and Biophysics, Weill Cornell Medicine, New York, NY 10021 USA; 5 The HRH Prince Alwaleed Bin Talal Bin Abdulaziz Alsaud Institute for Computational Biomedicine, New York, NY 10021 USA; 6 School of Medicine, New York Medical College, Valhalla, NY 10595 USA; 7 Universidade Estadual de Maringá, Nupelia/PEA, Maringa, Parana Brazil; 8 Unicesumar/Instituto Cesumar de Ciȇncia, Tecnologia e Inovação (ICETI), Maringa, Parana Brazil; 9 Biology Department, University of Illinois Springfield, Springfield, IL 62703 USA; 10 The Feil Family Brain and Mind Research Institute (BMRI), New York, NY 10021 USA; 11 Biological Sciences Department, NYC College of Technology, City University of New York, Brooklyn, NY 11201 USA

## Abstract

Modern metagenomic environmental DNA studies are almost completely reliant on next-generation sequencing, making evaluations of these methods critical. We compare two next-generation sequencing techniques – amplicon and shotgun – on water samples across four of Brazil’s major river floodplain systems (Amazon, Araguaia, Paraná, and Pantanal). Less than 50% of phyla identified via amplicon sequencing were recovered from shotgun sequencing, clearly challenging the dogma that mid-depth shotgun recovers more diversity than amplicon-based approaches. Amplicon sequencing also revealed ~27% more families. Overall the amplicon data were more robust across both biodiversity and community ecology analyses at different taxonomic scales. Our work doubles the sampling size in similar environmental studies, and novelly integrates environmental data (e.g., pH, temperature, nutrients) from each site, revealing divergent correlations depending on which data are used. While myriad variants on NGS techniques and bioinformatic pipelines are available, our results point to core differences that have not been highlighted in any studies to date. Given the low number of taxa identified when coupling shotgun data with clade-based taxonomic algorithms, previous studies that quantified biodiversity using such bioinformatic tools should be viewed cautiously or re-analyzed. Nonetheless, shotgun has complementary advantages that should be weighed when designing projects.

## Introduction

With the advent of next-generation sequencing (NGS), studies on DNA from environmental samples (environmental DNA or eDNA) have flourished. It is well known that inferences made from these studies can vary with the field, lab, and analytic techniques utilized^1, 2^. There are two principal ways that comparisons can be made when assessing the impact of NGS approaches on eDNA studies. The first entails comparison of sequencing platforms, such as 454 Roche vs. Illumina MiSeq using the same amplicon sequencing approach. The second compares sequencing approaches, the primary techniques being amplicon (sequencing all amplified products from a single gene; e.g., 16S) and shotgun (random sequencing across entire genomes). Several studies have performed such comparisons, with foci ranging from humans to studies of water and soil (Table 1).

**Table 1:** Summary of studies comparing different NGS sequencing strategies and sequencing platforms.

Strategy	Platform	Sample #	Target	Comment	Reference
A	Illumina HiSeq, MiSeq	24	Bacteria: Soil, human, and canine stool, mouth and skin	The results were very similar across lanes, read directions, and platforms (P < 0.0001), and also comparable to results obtained with the older GA-IIx. Increased sequencing depth did not provide additional information on beta diversity, but helped detect rare species. The HiSeq platform was recommended for large projects that aim at minimizing sequencing cost, while the MiSeq platform can give faster results for monitoring or preliminary studies.	Caporaso, J. G. *et al*. (2012)^19^
A	454, Illumina MiSeq	10	Eukaryotes (microbial): Soil	The two NGS approaches were extremely similar in the results they provided, especially for abundant amplicons.	Mahé, F. *et al*. (2015)^5^
A	454, Illumina MiSeq	7	Bacteria: Human stool, mouse, cow, leech, termites, sewage, mock	Reference-based operational taxonomic unit (OTU) clustering alone introduced biases compared to de novo clustering, preventing certain taxa from being observed. Low levels of dataset contamination were observed with Illumina sequencing. This cost-effective alternative to 454 was best when the same template primers, read merging, chimera checking, control libraries, and alternating indices between runs were applied.	Nelson, M. C. *et al*. (2014)^7^
A	Ion Torrent, Illumina MiSeq	3	Bacteria: Soil	The UniFrac distances between samples sequenced on both Illumina MiSeq and Ion Torrent were significantly correlated. “Differences between sequence technologies can be adjusted by adopting the correct pipeline of analysis”. The Q scores generated by different platforms were not directly comparable.	Pylro, V. S. *et al*. (2014)^9^
A	Ion Torrent, Illumina MiSeq	19	Bacteria: Human- derived, mock	The Ion Torrent platform had comparatively higher error rates and a pattern of premature sequence truncation specific to semiconductor sequencing. This led to organism- and direction-dependent biases provoking underrepresentation or failed identification of species.	Salipante, S. J. *et al*. (2014)^8^
A	454, Ion Torrent	17	Bacteria and Archaea: River sediments & oil sands tailings ponds	454 and Ion Torrent allowed for highly similar relative abundance estimates for major taxa and almost identical community structure patterns. Emulsion PCR limited amplicon size, which resulted in different forward primers being used. Apart from the following primer bias, “the 454 and Ion Torrent data sets were almost interchangeable, and both would have yielded the exact same ecological conclusions”. These ecological conclusions were based on physiochemical sediment data like clay and naphthenic acid values.	Yergeau, E. *et al*. (2012)^37^
A C	454 ﻿Sanger	6	Bacteria: Human dentition	454 resulted in significantly higher coverage estimates than the clonal analysis and provided a higher chance of finding rare species. Pyrosequencing, however, also significantly underestimated the relative abundance of Actinobacteria compared to culture.	Schulze-Schweifing, K. (2014)^4^
A ﻿C W	454, SOLiD Sanger SOLiD	1	Bacteria: Human stool	Sanger, 454, and SOLiD amplicon sequencing provided results comparable to the result based on SOLiD shotgun sequencing for overall community composition, but WGS sequencing allowed better identification of species.	Mitra, S. *et al*. (2013)^3^
A W ->W*	454, Illumina MiSeq Illumina GA-II, HiSeq	15	Bacteria: Soil	The small subunit (SSU) extracted from the shotgun approach yielded higher diversity estimates than straight amplicon methods, both taxonomy- and OTU-based (mainly due to primer bias and chimeras in amplicon sequencing). On the other hand, samples were clustered in similar ways using the two approaches. Another advantage of shotgun sequencing was that it allowed the calculation of the fungus/bacteria ratio, which is an important measure of soil health. The large subunit﻿ (LSU) rRNA gene provided even better phylogenetic resolution than SSU.	Guo, J. *et al*. (2015)^2^
A ﻿W	Illumina MiSeq Illumina HiSeq	1 each	Bacteria: Hot spring water thermophiles	Amplicon and shotgun sequencing allowed for comparable phylum detection, but shotgun sequencing found more. The 16S rarefaction curve indicated that a fraction of the species diversity remains to be discovered. Complete functional groups were missed by this approach, like thermophile denitrifying bacteria.	Chan, C. S. *et al*. (2015)^13^
A ﻿W	Ion Torrent, Illumina MiSeq﻿ Ion Torrent, Illumina MiSeq, HiSeq	6	Bacteria: Human stool	Changing sequencing methods and informatics approaches to binning sequences to taxa had the greatest impact on variance in the analysis – greater than the difference in between samples. Compared to amplicon sequencing, WGS approaches increased the information gained and reduced biases, but had their own issues mainly related to sequencing depth and read length. While HiSeq offered a much greater sequencing depth that allowed the detection of rare species, the high species count might have been inflated due to misalignments of short reads. At the same time, it performed worst in predicting genes. Ion Torrent generally showed an intermediate performance.	Clooney, A. G. *et al*. (2016)^14^
A W	Illumina HiSeq Illumina GA-II	16	Bacteria: Soils (deserts, tundra, forests)	The two methods yielded nearly identical estimates of the overall differences in soil bacterial community diversity and composition. The study showed clear limitations of shotgun sequencing depth, that only 13–23% of reads could be annotated, and many of these were misannotated. Still, “for certain questions, shallower sequencing of many samples may be more useful than deeper sequencing of fewer samples”.	Fierer, N. *et al*. (2012)^20^
A & W	Illumina MiSeq	16	Bacteria: Kefir, human stool, mouse stool, mock mix	Shotgun metagenomics offered a greater potential for identification of strains, which still remained unsatisfactory. It also allowed increased taxonomic and functional resolution, as well as the discovery of new genomes and genes.	Jovel, J. *et al*. (2016)^15^
A ﻿W	454, Illumina MiSeq 454, Illumina HiSeq	4 to 10, depending on comparison	Bacteria: Marine plankton	Metagenomic approaches were reported to have an advantage over amplicon approaches. They rendered more truthful community richness and evenness estimates by avoiding PCR biases, and provide﻿d additional functional information. While both platforms “presented a good agreement by recovering taxa from the same evolutionary groups” when comparing metagenomic shotgun sequencing, many more unique genera were recovered with Illumina than with 454 sequencing. This was partly due to better detection of rare taxa.	Logares, R. *et al*. (2014)^24^
A ﻿W	454 Illumina GA-II	4	Bacteria: Freshwater	Taxonomic composition of each 16S rRNA gene library was generally similar to its corresponding metagenome at the phylum level. At the genus level, however, there was a large amount of variation between the 16S rRNA sequences and the metagenomic contigs, which had a tenfold resolution and sensitivity for genus diversity.	Poretsky, R. *et al*. (2014)^10^
A ﻿W	Illumina MiSeq Illumina HiSeq, MiSeq	1	Bacteria: Human stool	Whole genome sequencing approaches “enhanced detection of bacterial species, increased detection of diversity and increased prediction of genes”. The MiSeq platform provided better de novo contig assembly and species detection with its longer reads.	Ranjan, R. *et al*. (2016)^11^
A W	454 Illumina GAIIx	51	Bacteria: Human Microbiome Project, vaginal microbiomes	The developers of the Metagenomic Phylogenetic Analysis tool MetaPhlAn showed that it was advantageous to comparable tools. They further underlined the advantages of analyzing taxonomically specific marker genes selected from WGS data (~4% of genes) over amplicon approaches, by “enabling efficient, high-resolution taxonomic profiling”. Yet, while they reported better statistical support for metagenomic sequencing (~10^8^ as compared to <10^4^ reads/sample), the advantages were not evident from their data, as the results for relative abundances of genera were “remarkably similar in all clusters”, and they did not include species level results for amplicon data.	Segata, N. *et al*. (2012)^30^
A ﻿W	454 454, Illumina HiSeq	3	Bacteria and Archaea: Synthetic communities of 64 sequenced species	“Both Illumina and 454 metagenomic data outperformed amplicon sequencing in quantifying the community composition, but the outcome was dependent on analysis parameters and platform.” Metagenomic sequencing outperformed most SSU rRNA gene primer sets, with V13 recovering the best accuracy. Archaea had distinct biases to Bacteria.	Shakya, M. *et al*. (2013)^38^
A: SSU, LSU, ITS W	Illumina MiSeq Illumina HiSeq	14	Fungi: Soil	The metagenomic shotgun and amplicon approaches performed similarly for identification of most fungal classes. WGS was far inferior in detecting OTUs and identifying species than the amplicon approach using internal transcribed spacer﻿s (ITS) as an amplicon target. This was largely due to low (0.005% of DNA) and uneven recovery of fungal rDNA sequences, and lacking fungal data in the reference databases. This “identification bias” was very difficult to quantify or compare among studies.	Tedersoo, L. *et al*. (2015)^12^
W	454, Illumina GA-II (HiSeq)	1	Bacteria: Freshwater planktonic community	The two platforms performed similarly as 90% of the microbial taxa from the two methods overlapped and the abundance of taxa as determined by the two approaches was highly correlated (R^2^ = 0.9). While Illumina recovered longer & more accurate contigs and 14% more complete genes; pyrosequencing might be superior for resolving sequences with repetitive structures or palindromes, and for metagenomic studies based on unassembled reads. Illumina HiSeq seemed to perform similarly to GA-II.	Luo, C. *et al*. (2012)^6^
W	PacBio RS, Ion Torrent, Illumina GA-IIx, HiSeq, MiSeq	4 genomes	Bacteria: 4 species	Pacific Biosciences RS needed far more DNA, but may be useful for studies focused on de novo sequencing, alternative splicing or epigenetics. It featured read lengths an order of magnitude higher than the other platforms (average: 1500 bases) and insert sizes of up to 10 kb. This read length combined with a very high raw error rate of 13% led to 0% of reads being error-free (75% and 15% for Illumina and Ion Torrent, respectively), which complicated single nucleotide polymorphism (SNP) calling. The errors were evenly distributed, though, while Illumina had higher error rates after long homopolymer tracts and the GGC motif. Ion Torrent failed at sequencing homopolymer tracts, had strand-specific errors, and severe coverage bias for AT-rich genomes.	Quail, M. A. *et al*. (2012)^39^

In the Strategy column abbreviations are A = 16S amplicon, C = clonal amplification, W = WGS shotgun, and W* = WGS where SSU sequences are extracted and used.

The results of prior comparative studies regarding eDNA sequencing vary (Table 1). When Sanger methods are compared to 454 and SOLiD, these approaches perform comparably^3, 4^. Illumina and 454 platforms also behave similarly^2, 5–7^. In contrast, for amplicon strategies, higher error rates are found with the Ion Torrent due to premature sequence termination, as compared to Illumina^8^. However, the right choice of analysis pipeline can sometimes ameliorate differences between these sequencing technologies^9^.

By far the most common comparison is shotgun vs. amplicon^10–15^. Shotgun approaches regularly infer more diversity than amplicon^2, 10, 11, 14^. These studies use sample sizes ranging from 1–51, with an average of 11 samples (Table 1). In the present study we compare amplicon and shotgun analyses of 49 samples from across the principal river floodplain systems in Brazil^16^. Ecological metadata associated with all 49 samples allows us to compare the impact of sequencing platform on ecological interpretations, which has only minimally been explored previously.

## Results

### Overall Taxonomic Comparison

Amplicon sequences were classified into 20 phyla while shotgun sequences were classified into only nine. Eight of the nine phyla recognized by shotgun were also recognized from amplicons. Deinococcus-Thermus is the only unique phylum to the shotgun results and is only detected at one site out of 49. Furthermore, it was not found in global comparisons of freshwater bacteria^16, 17^, suggesting that further exploration is needed to determine if this is a false positive.

**Figure 1:**
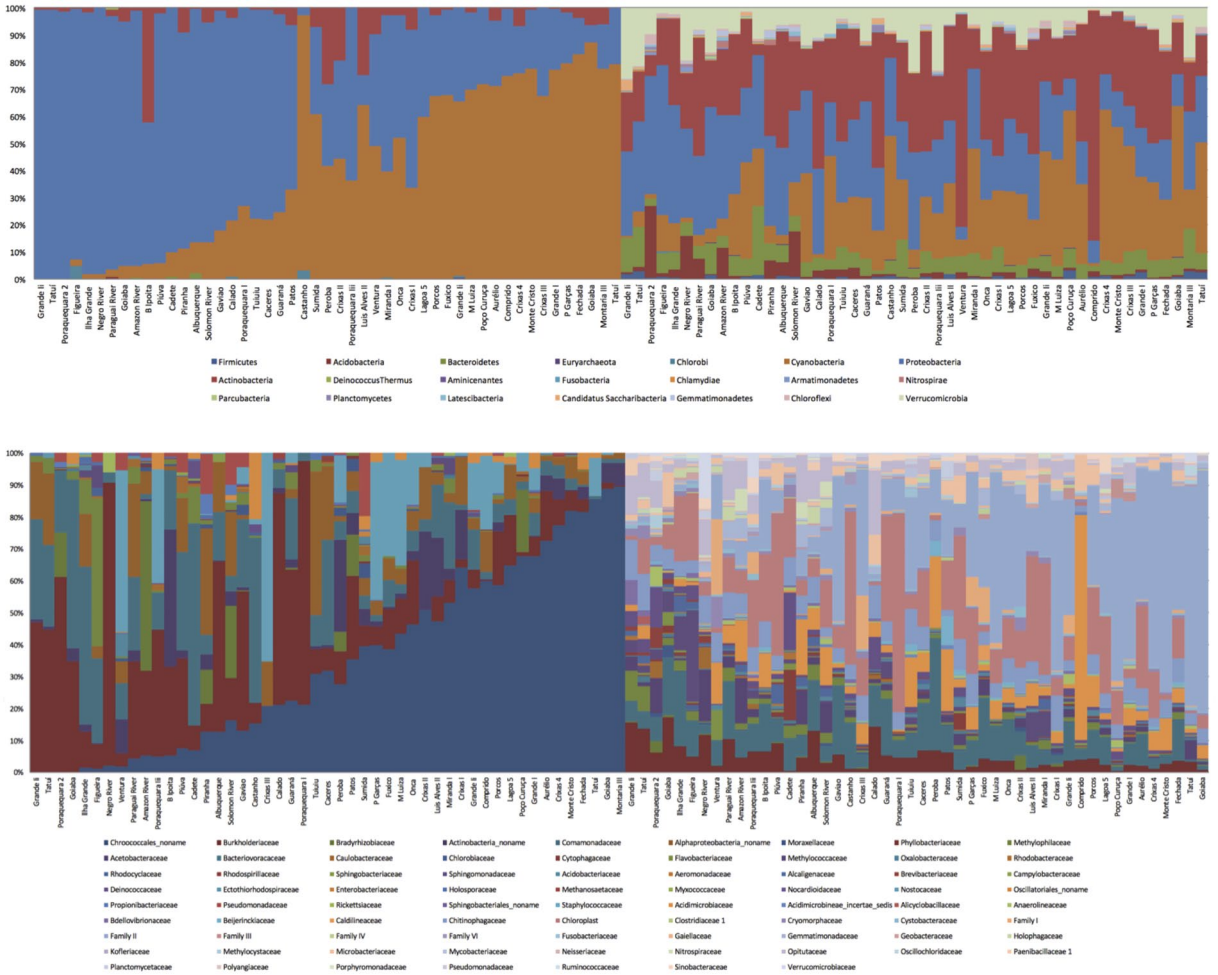
Bar plots showing the proportion of reads of the 49 samples in this study at the phylum (top) and family (bottom) levels, comparing the shotgun (left) and amplicon (right) techniques. The samples in both phylum-level and family-level panels are sorted on the percentage of Proteobacteria and Cyanobacteria Family II, respectively, in the shotgun data set.

Figure 1 shows strongly contrasting proportions of phyla per sample for the two sequencing approaches, with only two phyla dominating the shotgun identifications. While both approaches had sequences classified as Bacteroidetes, the amplicon approach detects higher proportions of this phylum. This bias is also seen for Actinobacteria and Firmicutes. The most evident similarity of the two approaches is that Proteobacteria, Actinobacteria, and Cyanobacteria are found as major components of the identified sequences.

Results of the family-level classification reveal even less overlap between the two approaches. The amplicon approach results in classification of 56 families, while the shotgun approach recognizes 41 families, but only 18 families show overlap between the two strategies. Differences in the percentages of families detected using the two sequencing approaches are striking (Fig. 1). Several families, however, are similarly recognized as major components of each sample by the two approaches - Burkholderiaceae, Comamonadaceae, and Methylophilaceae.

There are over 50 phyla represented in the whole genome database, which currently contains over 83,000 fully sequenced prokaryotic genomes. These genomes show the upper extent of species representation that would be available for searching in any of the currently available classification programs. We examined the distribution of phyla found in our samples for both the amplicon and shotgun approaches in the context of these fully sequenced genomes. Supplementary Figure 1 shows the phyla available in the National Institutes of Health (NIH) genome database, while highlighting those found using either amplicon or shotgun sequencing that are also in this database. Of the 20 phyla we identified in the amplicon based study, 16 have phyla members with whole genomes sequenced in the NIH database. The four phyla identified using the amplicon approach that do not have whole genome sequences in the NIH database are Aminicenantes, Latescibacteria, Parcubacteria, and Saccharibacteria. All nine shotgun-identified phyla have representatives with whole genomes sequenced (17 phyla have data available for ﻿MetaPhlAn).

**Figure 2:**
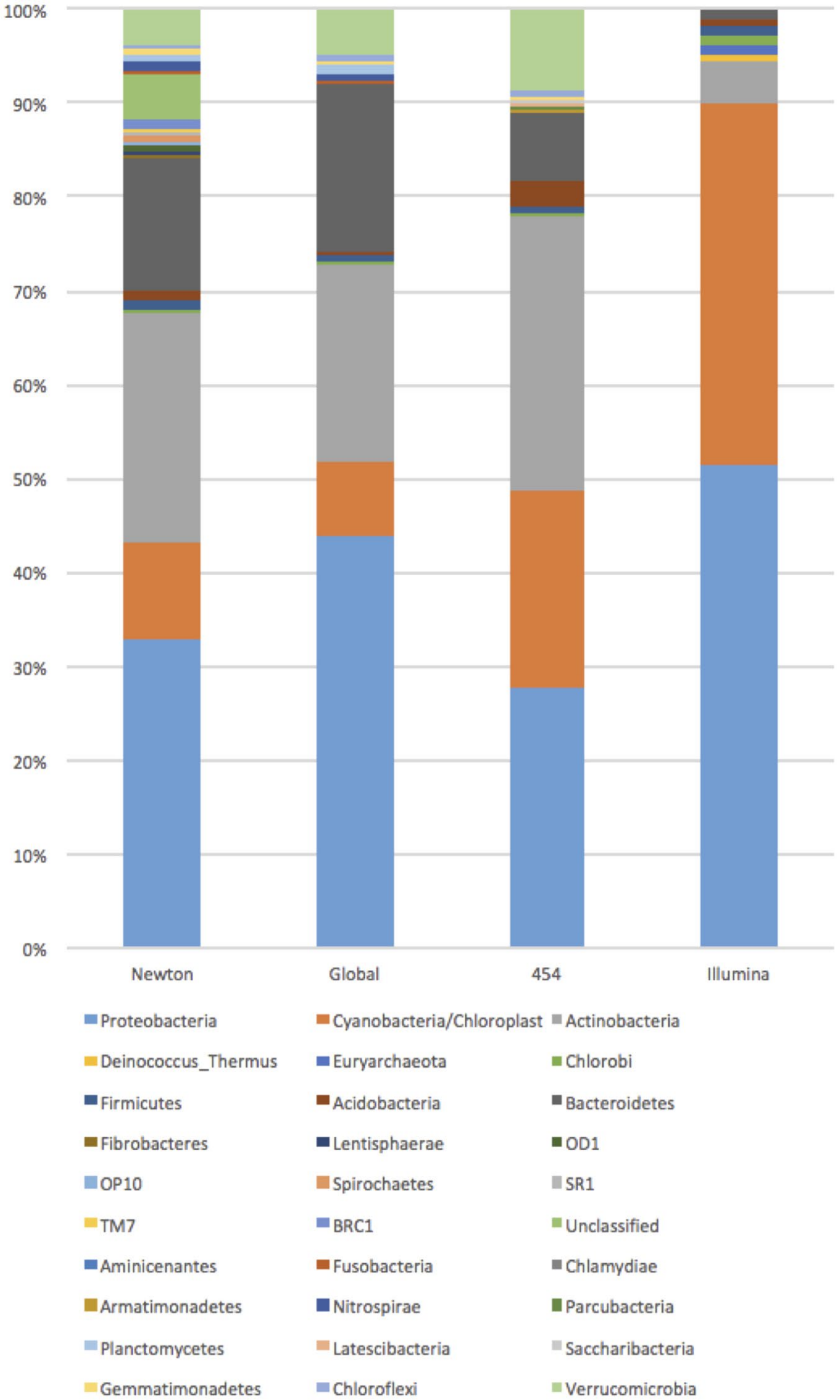
Bar plots of the summed proportion of reads (on Y axis) at the phylum level for the two sequencing strategies we have used to review Brazilian sites (labeled as 454 amplicon and Illumina shotgun) and the global comparisons of the meta-analysis^17^ (Newton) and our prior global amplicon comparisons^16^ (Global). The color code for phyla in the plots is given at the bottom of the figure. Taxonomic nomenclature follows that in the RDP. Note that several of these phyla have since been formally named: TM7 = Saccharibacteria; SR1 = Absconditabacteria; OP10 = Armatimonadetes; OD1 = Parcubacteria.

We next compared the overall composition of phyla in the 49 samples for shotgun and amplicon-derived sequences to global datasets of lake bacteria (Fig. 2). We found strong congruence between the amplicon results and those from all prior amplicon research across the globe. While there is overlap in some of the major phyla in lake systems, the shotgun approach detects different proportions of these phyla that are dissimilar to known freshwater systems. Specifically, the shotgun approach detects higher proportions of Proteobacteria and Cyanobacteria than the amplicon approach conducted here, the Newton study^17^, and our prior global amplicon-based comparison^16^, with the exception being somewhat similar levels of Proteobacteria in our prior global amplicon comparisons.

### Impact on Ecological Inference

**Figure 3:**
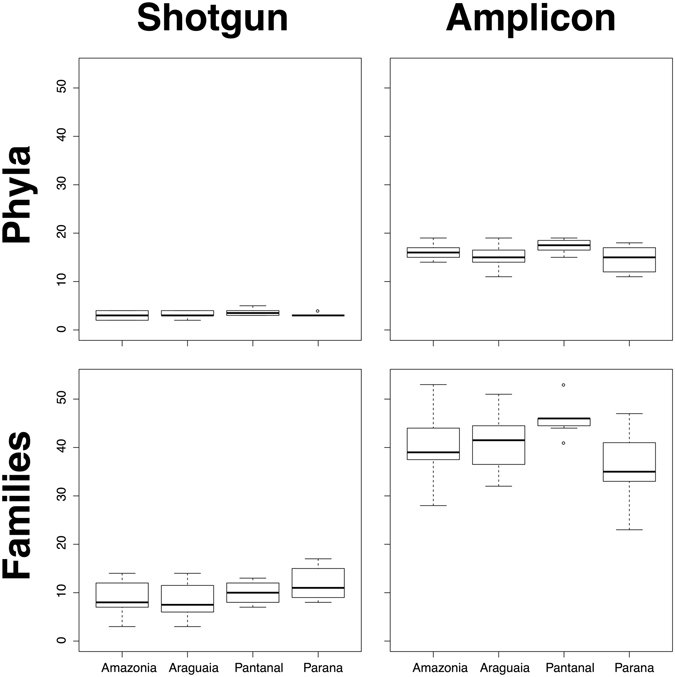
Box-and-whisker plots of shotgun vs. amplicon sequencing strategies showing taxon richness at the phylum and family levels. Boxes are middle quartiles divided by the medians, whiskers are 1.5x the interquartile range, and dots are outliers.

In order to assess the impact of the two sequencing strategies on ecological inferences, we compared both datasets using a variety of standard comparisons used in community ecology, focusing on taxonomic richness, taxonomic abundance, and community composition. Our simplest comparison for sequencing strategy – box and whisker plots of taxonomic richness across each of the river floodplain systems – revealed clear differences (Fig. 3). Each river floodplain system had lower taxonomic richness from shotgun sequencing, which corresponds with the overall richness findings mentioned above. However, more notable is that in the amplicon results, the Pantanal stands out based on taxonomic richness. This pattern is not recovered with shotgun sequencing. In fact, shotgun sequencing at the family level hints at the Paraná being slightly richer.

**Figure 4:**
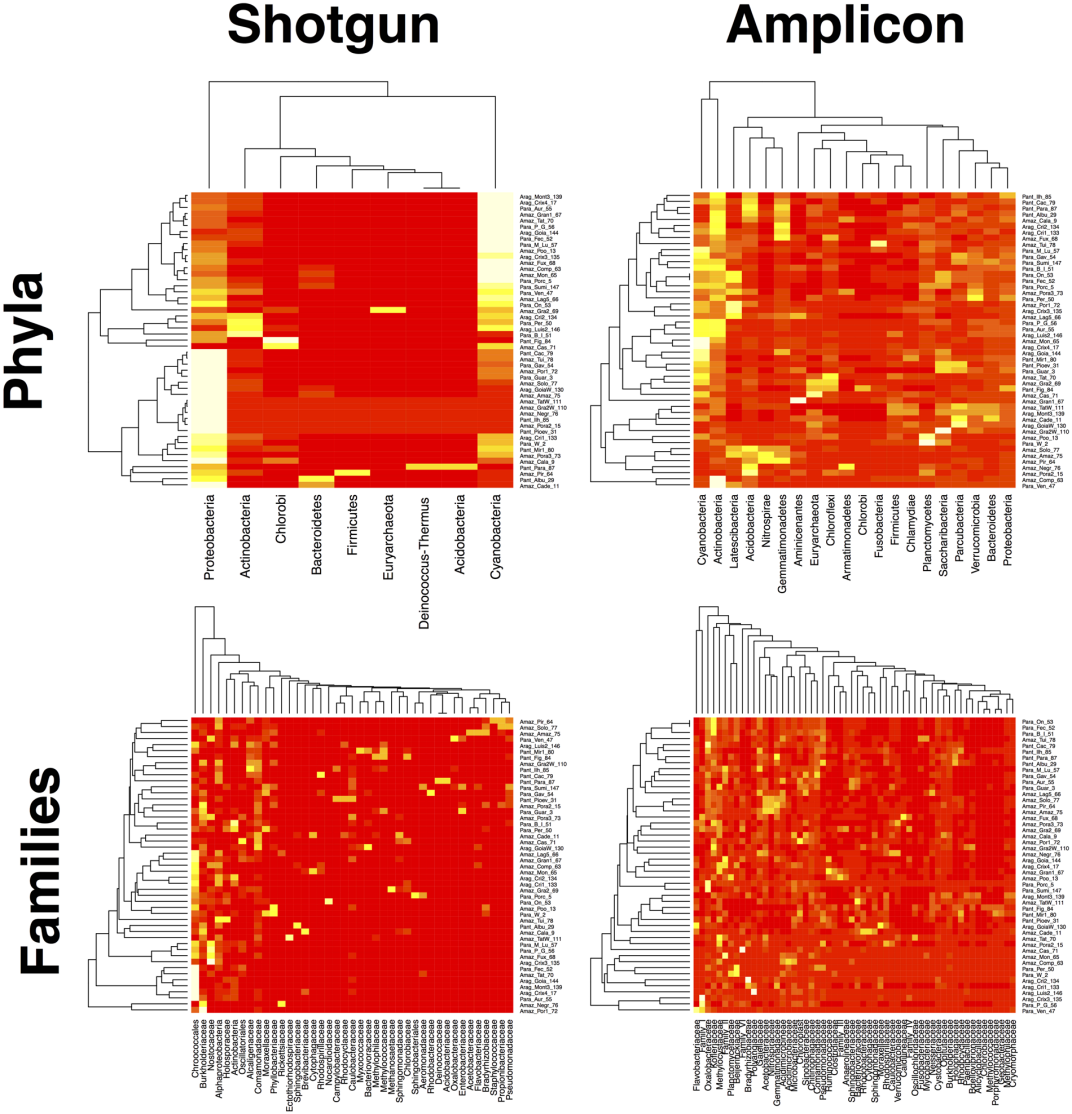
Heatmaps of shotgun vs. amplicon sequencing strategies showing taxon abundance at the phylum and family levels. Please note that data were double standardized for better visualization of low abundance taxa and because this is common for ordinations.

Heatmaps show the abundance of taxa at each site to be more homogenous in shotgun sequences (Fig. 4). This is partially a reflection of fewer taxa being found with this method, as noted in the comparisons above. However, Cyanobacteria and Proteobacteria at the phylum level particularly drive this pattern, as is further reflected by the accompanying cluster diagrams.

**Figure 5:**
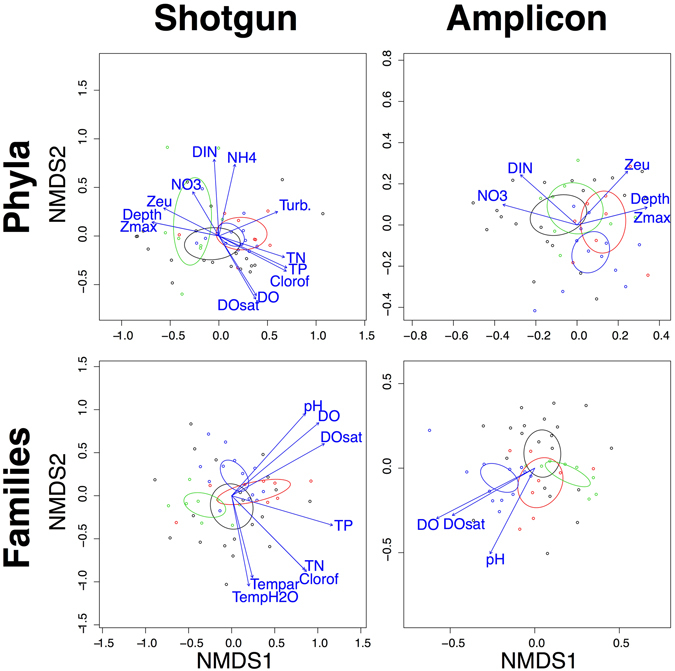
NMDS plots for datasets from the shotgun and amplicon techniques for the family and phylum level. Color codes for sites and confidence ellipses are as follows: black = Amazon, red = Araguaia, green = Pantanal, and blue = Paraná. Blue arrows indicate environmental variables that correlate to ordinations. See Supplementary Table 1 for a list of expanded environmental variable names.

Nonmetric multidimensional scaling (NMDS) analyses for both the amplicon and shotgun approach do not result in any of the four river floodplain systems in the study being particularly distinct (Fig. 5). The environmental variables significantly corresponding with the ordinations are only somewhat similar between amplicon and shotgun approaches both in terms of which variables are significant and how they align with the ordination (Supplementary Table 1). Notably there are many more environmental correlates in the shotgun dataset, while variables significant for amplicon-generated sequences represent a subset of those found with shotgun.

**Figure 6:**
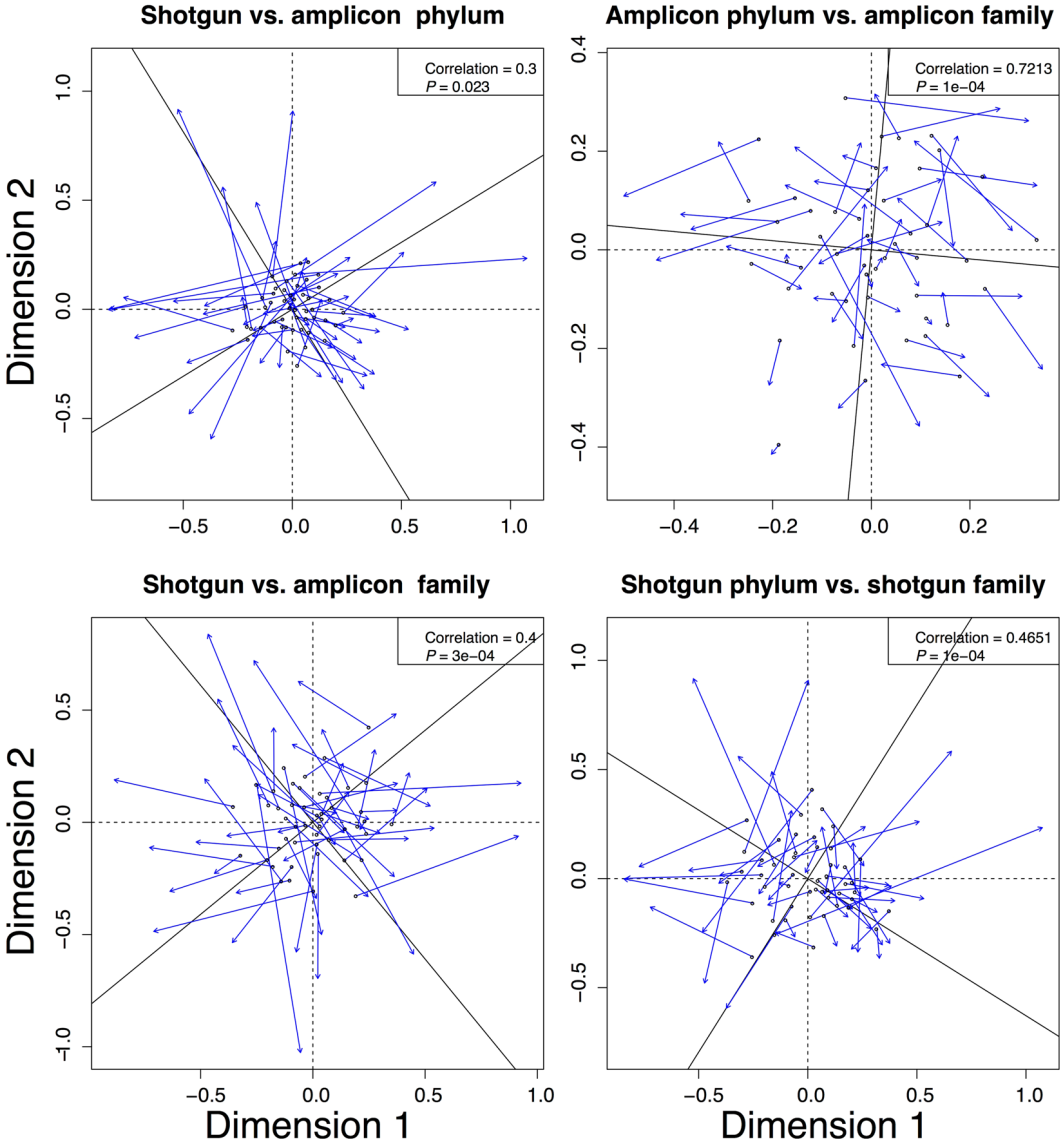
Procrustes visualizations of NMDS plots compared at the phylum and family levels for the amplicon vs. shotgun approaches, as well as comparisons of amplicon or shotgun at both taxonomic levels. Test statistics for Procrustes tests are presented for each comparison.

Figure 6 depicts Procrustes tests of the NMDS ordinations produced for the two data sets: significant correlation of the sequencing strategies was found, but it was weak given their use of the same extracted DNA. The finer scale family level comparisons are more similar, despite the actual taxa named for each sequencing strategy at the family level having poor overlap.

To get a further sense of the quality of the datasets and to compare the strength of their correspondence to one another, we compared each dataset (amplicon or shotgun) to themselves using the phylum and family level identifiers. This showed much stronger congruences for the amplicon dataset than the shotgun dataset. The correlation was only slightly stronger for the shotgun comparison than it was for the family level comparison of shotgun vs. amplicon, whereas the amplicon comparisons at the phylum and family level were about twice as strong as the comparisons between sequencing methods.

### Quality Assessments of Analyses

**Figure 7:**
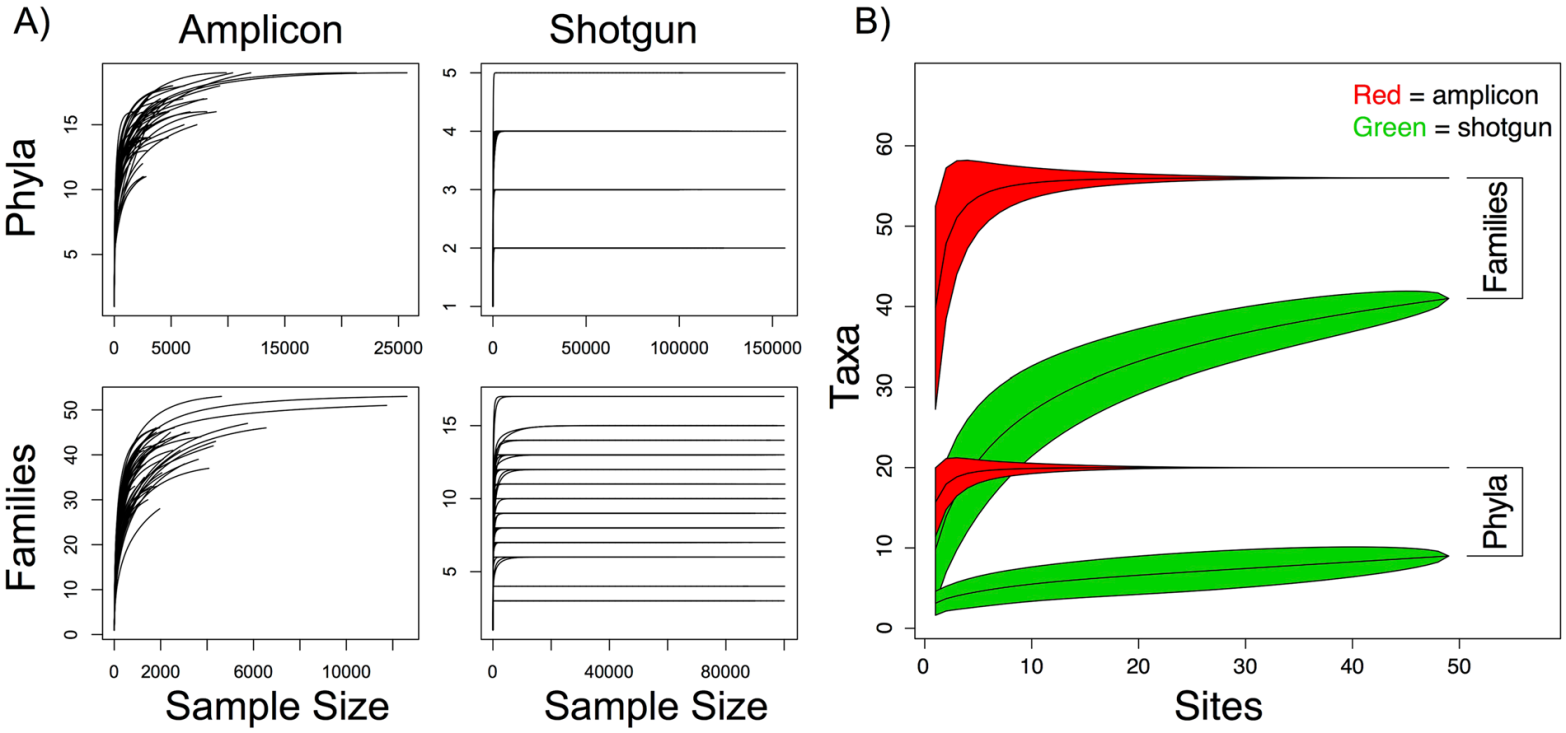
Comparisons of the sampling efforts for amplicon and shotgun sequence data at the phylum and family levels using (**A**) rarefaction curves for individual sites and (**B**) species accumulation curves for the 49 total sampling sites in Brazil.

Our comparisons verifying the quality of our data showed our sampling was thorough. Following QC of the 454 GS Junior generated sequences, 346,042 reads were moved downstream. This number is only a small fraction of the reads generated by the Illumina HiSeq 2500, which, after QC, was ~575 M reads (averaging 12 M pairs of 125 × 125 bp reads per sample). Despite the discrepancies in read count, on a per site basis, it was clear that rarefaction curves reached their asymptotes consistently, indicating that read depth was likely sufficient for both methods, given these taxonomic classifiers (Fig. 7A). The asymptotes are higher and more consistent in taxon richness for the amplicon data. For example, the shotgun data for both taxonomic levels revealed approximately one third of the taxon richness found from amplicons, corroborating our comparisons of taxon richness above.

These rarefaction results are further borne out by the species accumulation curves, where both methods at each taxonomic level have generally reached their asymptote (Fig. 7B). The amplicon data reached full asymptotes with around 10 sites, showing that the method has robust taxonomic sampling even for small numbers of sites. In contrast, the shotgun asymptotes never fully level out, indicating that a large number of sites would be necessary to have a robust taxonomic sample. This is further indicated by our estimates of true taxon richness, which found that, while still lower than amplicon richness, true taxon richness is notably higher with shotgun than could be found with the total sites used in this study (Supplementary Table 2); also note the predictions for shotgun data have a high degree of uncertainty.

## Discussion

Our study compares the efficacies of the two NGS sequencing strategies used for eDNA studies (amplicon vs. shotgun) over one of the largest datasets of environmental samples to date. We found the amplicon approach was far more discerning in almost all respects, contrasting general dogma in the field and all but one of eleven empirical studies in Table 1 that compared these strategies. Unlike our study, that contrasting study differed primarily because of issues with fungal rDNA recovery and deficient databases, rather than due to the systematic biases of the method^12^.

Our study showed weak correlation between the two methods, indicating that while taxonomic overlap exists at both the phylum and family levels the methods are substantially different. Under half the phyla identified from amplicons were found with shotgun; almost all of the phyla recognized by the shotgun approach were also recognized by the amplicon approach. About 30% fewer families were identified from shotgun. This superior performance from amplicons comes despite having <1% of the total reads produced from shotgun. The amplicon results were also far more consistent with prior research on the biodiversity of freshwater systems (Fig. 2). In addition, the Procrustes tests indicated that there is only weak correlation for community composition between the two sequencing strategies using NMDS.

The key difference between the amplicon and shotgun derived data in our study was taxonomic breadth and abundance, whether looking at the overall results or site-by-site. The lower taxon counts for shotgun sequencing appear to be due to issues inherent to the shotgun technique, as well as to the database size. As genome databases are continuously improving and expanding in size, this problem should become less significant. New approaches in multi-enzyme and mechanical shotgun extraction and sequencing techniques may also help^18^. Additionally, shotgun sequencing is complicated by having many reads map to unknown species, which reduces the number of taxonomically-applicable reads (often the majority of reads), and this issue may be more problematic in complex environments such as river basins.

The fundamental issue with the shotgun technique was that taxon richness reached an asymptote on a per site basis at low and unpredictable levels, as compared to amplicon results (Fig. 7A). While this high degree of variability can potentially be overcome by using a large number of sites (note the high variance in total predicted taxa in Supplementary Table 2 and the longer asymptote in Fig. 7B), this is not particularly helpful, as it is a fundamental goal in biodiversity studies to get at the true richness and abundance of organisms at each individual site. Yet, the environmental correlates were greater with the shotgun data (more below). The rarefaction asymptotes of Fig. 7A indicate that further sequencing is unlikely to provide additional insight on a per site basis, at least when using MetaPhlAn2. In contrast, some studies have shown that a greater sequencing depth can be useful for the detection of rare species; unfortunately, it generally comes at the cost of shorter reads that are frequently misaligned - a process that leads to an inflation of both species count and diversity estimates^4, 14, 19^.

As for genomic databases, even for microbes, they are in their infancy^11, 12, 20^. While genomes deposited in these databases are increasing at an astonishing pace, they have a long way to go^15^. This is especially true when compared to the well-curated 16S microbial databases like RDP^21^, SILVA^22^, and Greengenes^23^. This appears to be less of a problem in studies on well-characterized systems like the human microbiome (Table 1).

By definition, all nine of the phyla recognized in the shotgun dataset have whole genome sequences in the database. On the other hand, the 20 amplicon phyla determinations use 16S rDNA sequences to make the identifications, so not all of them necessarily have sequences in the whole genome database. Indeed, only 80% of the phyla identified using the 16S amplicon approach also have whole genomes sequenced from members of those phyla (Supplementary Fig. 1), leaving us with only a minor taxonomic overlap between databases. This discrepancy at the phylum level clearly entails a massive lack of resolution at finer taxonomic levels (e.g., for families reviewed here). Missing a single phylum is disconcerting, let alone 20% of phyla.

Given the 16S vs. genome database discrepancy, many shotgun sequences are surely assigned to inappropriate taxa. These incorrect IDs are most likely close relatives of taxa that have sequenced genomes. Thus, the IDs may still have some merit based on the fact that closely related taxa generally have phylogenetically constrained traits that make them more similar (ecologically, physiologically, etc.) to one another than to more distant relatives^24^. However, ecological analyses using higher taxa as surrogates for species achieve variable results depending on the types of input data^25^. In microbial communities, functional diversity cannot be directly predicted from phylogenetic diversity. For example, while in the macroscopic world it is an accepted paradigm that an ecosystem with a low level of taxonomic richness will also have a reduced functional diversity, this does not seem to apply to microbial communities^20^.

Because of the putative cases of mistaken identity with shotgun sequencing, we chose not to use UniFrac or any of its derivative distances (e.g., weighted and generalized; see ref. 26) for community level analyses. For microbial eDNA community ecology, multivariate analysts now generally favor these phylogenetically adjusted measures rather than simply considering taxa as independent entities. However, without highly accurate identifications, accounting for a specific phylogeny makes little sense: recall that only half the amplicon-recovered phyla were found with shotgun, indicating that many shotgun sequences were identified to incorrect phyla - a phylogenetically gigantic distance.

The biases of close, but not exact, identifications are almost surely less extreme when considered as fully independent entities (i.e., not using UniFrac, but more traditional non-phylogenetic distance matrices). Considering taxa as fully independent entities is standard for community ecology of large eukaryotic organisms. Yet, despite the acceptability of both methods, it is still a notable difference that shotgun data should not – in our opinion – rely on phylogenetically accountable methods until the databases become larger and the tools more sensitive.

Throughout our study we focused on commonly used bioinformatic pipelines. While the RDP appears to work well for amplicons, our findings of MetaPhlAn having lower quality results for shotgun could be called into question. However, MetaPhlAn is one of the most popular taxonomic categorizers; for instance, it was used in the Human Microbiome Project^27^. More importantly, it relies on clade-specific marker genes, which is crucially important for accurate identifications in bacterial biodiversity studies and is a common algorithmic approach. We believe that current practices for analyzing shotgun data that do not use clade-specific markers may be inappropriate for bacterial taxonomic identifications. Future studies should compare less conservative approaches, such as PhyloSift^28^.

Due to conjugation, horizontal gene transfer is rampant in bacteria. It is equally well established that there is a core set of genes across bacteria that are highly conserved and rarely transferred; this is generally referred to as the core genome^29^. While amplicon-derived analyses take advantage of a single gene in the core genome, shotgun relies on genes across the entire genome. Accordingly, the analytics of shotgun will inevitably lead to avoidable misidentifications if based around genes not found in the core genome. This is a major problem for biodiversity and ecology studies, as confident identifications are paramount. Future shotgun analytics can therefore benefit from limiting taxonomic identifications to sequences from the core genome or clade-specific marker genes (as done by MetaPhlAn^30, 31.^

Furthermore, while our results could be confounded by the fact that we sequenced amplicons via 454 and shotgun via Illumina, we found the majority of studies in Table 1 comparing the amplicon procedure for 454 vs. Illumina agree that these sequencing platforms give highly similar results. Additionally, while Illumina is the dominant NGS platform, amplicon and shotgun studies generally use different Illumina platforms to meet their goals (e.g., HiSeq and MiSeq, respectively; see Table 1). Thus, we believe that our results and comparisons are valid. It is also worth noting that if there were to be an issue with one of these sequencers, it would be assumed that it would be the 454, as it had fewer than 1% of the reads sequenced for Illumina (as expected) - making our results akin to a fisherman with a single fishing rod catching more fish than a commercial trawler.

The only result that is agnostic towards (or at least difficult to interpret for) shotgun or amplicons was in regards to the environmental correlates of the NMDS ordinations (Supplementary Table 1, Fig. 5), which found shotgun to have more significant variables associated with certain metadata. While this could be in favor of shotgun, it is unlikely as the input matrix was so depauperate in terms of taxon richness and evenness across taxa. More likely, this result could be due to a more simplified ordination space that is largely driven by clear divisions by site for a few taxa, as exemplified by the heat maps (Fig. 4). More correlates were found for the phylum level in both sequencing methods, further supporting the idea that the ordinations driven by fewer taxa could be increasing the number of correlates we found. It is also worth noting that for more thoroughly researched microbial floras that have many genomes sequenced, the shotgun system may outperform the amplicon-based approaches as it will provide useful data for a larger array of questions. This already might be the case for urban environments or the human microbiome^32^.

While both amplicon and shotgun sequencing methods have their own advantages for microbial studies, amplicon sequencing was clearly superior for the goals of microbial eDNA community ecology in the reviewed lakes of floodplain systems from Brazil. Further studies should strive for comparisons of even larger datasets across a greater number of habitats, as there can be major differences in conclusions drawn based on the type of sequencing conducted^33^. At this point, any large scale studies should at minimum conduct pilot comparisons between these techniques to choose the more appropriate option.

## Methods

### Sample Collection and DNA Isolation

The samples compared in this study were analyzed with the 454 amplicon approach in a previous publication^16^ and detailed information on the collection of the samples can be obtained from that publication. We used the DNA isolated from the water samples in our prior work^16^ for comparative sequencing with Illumina-generated shotgun data. Specifically, we matched 49 of the amplicon sequenced samples from our prior study (58 total) with the shotgun data generated here. The list of samples is provided in Supplementary Table 3. Environmental data were also recorded for each site, as detailed in our prior work^16^.

### Amplicon Library Preparation

454 library construction, primer design targeting a specific segment of the 16S rRNA gene (per the Earth Microbiome Project), and work up of amplicons (i.e., amplification and sequencing) are as detailed in our previous work^16^.

### Shotgun Library Preparation

DNA fragments were prepared into sequencing libraries according to modified manufacturer’s standard protocols, using the TruSeq Nano DNA library preparation protocols (FC-121-4001) and the QIAGEN Gene Reader DNA Library Prep I Kit (cat. no. 180984). 50–100 ng of sample DNA went through Covaris fragmentation to ~500 nucleotides. AMPure XP beads were used for size selection (removal of small fragments <200 bp) and removal of excess reagents. DNA was end-repaired to create blunt ends on both 3′ and 5′ ends. Then A-tailing, or the addition of dATP to the 3′ end, was carried out, which increases the stability of the DNA fragments, prevents concatamer formation, and enables ligation to occur with a complementary T nucleotide found on indexes. Next, the DNA fragments were tagged with index ligation tags. Eighteen cycles of Polymerase Chain Reaction (PCR) were then used to amplify sample DNA fragments. An AMPure XP bead wash was then used to purify DNA libraries. Fragments were visualized on a BioAnalyzer 2100 to check quality and average nucleotide length and concentration was measured by Qubit quantification (ng/uL).

### Sequencing

Using HiSeq (v4) SBS chemistry, we multiplexed 24 samples per lane on a HiSeq 2500 and processed the raw data using the Illumina RTA software and CASAVA 1.8.2. All samples were then checked for standard CASAVA QC parameters (all reads pass filter). Specifically, all samples had high (>Q20) quality values at the median base, low percent alignment to PhiX (<1%), and similar insert size (550 ± SD of 70 bp).

### Sequence Trimming and Quality Control

The amplicon analysis pipeline is described in our prior work^16^. Concisely, we used a multi-tiered approach to assure the quality of downstream sequence data. We demultiplexed the sequences and implemented five standard 454 quality filters on the GS Junior (Dot, Mixed, Signal Intensity, Primer and TrimBack Valley). Thereafter, sff_extract (http://bioinf.comav.upv.es/sff_extract/index.html) was used to create .fasta, .fasta.qual, .fastq, and .xml files. Low quality reads were removed and key/adaptor sequences were clipped using sff_extract. The results of this filtering were visualized using FastQC (http://www.bioinformatics.babraham.ac.uk/projects/fastqc/). Two binaries, FASTQ/A Trimmer and FASTQ Quality Trimmer (part of the FASTX toolkit; http://hannonlab.cshl.edu/fastx_toolkit/), were used to further trim low-quality regions. The final data set consisted of sequences with bases having a Phred quality score ≥ 25.

### Taxonomic Classification of Sequences

Diversity at the family and phylum levels for the 454 data set was assessed as in our prior work^16^. Succinctly, we used the RDP categorizer to obtain classifications at broad (phylum) and narrow (family) levels of taxonomic diversity; please see the Discussion section and our prior work^16^ for an explanation of why finer (i.e., genus and species level) taxonomic resolution may be inappropriate. Over 50 phyla and 350 families are assessed by the RDP categorizer^34^. MetaPhlAn (v2.0)^30, 31^ was used to analyze the shotgun data. Samples were run with the –ignore viruses parameter to filter out reads matching to phiX that is spiked during some library preparation procedures and becomes a contaminant in the microbiome analysis.

### Comparisons of Amplicon and Shotgun Sequences

Results from each method were summarized in several formats. Percentages of reads by taxon were visualized for both phyla and family levels. Since our samples are from lakes of floodplain systems, we compared their taxonomic distributions to a major survey of lake microbiota^17^ as well as our prior survey of freshwater microbiota^16^. Heatmaps with site and taxon cluster diagrams were produced for each method using the “heatmap” function in R^35^. Species richness was calculated and visualized with box-and-whisker plots in R. To compare the sequence quality in further detail, we produced species accumulation curves (using “specaccum”), rarefaction curves (using “rarefy”), and estimates of true taxon richness (using “specpool”) in R with the vegan package^36^.

To compare community level differences between those taxa identified with each sequencing method, NMDS ordinations were constructed using function “metaMDS” from the vegan package in R^36^; default settings were used except trymax = 10,000. For simplicity between comparisons, two-dimensional ordinations were selected. Environmental vectors were fit to the ordination results using “envfit” (vegan). Separation of floodplains was tested with PERMANOVA analyses conducted with the “adonis” function (vegan). Non-randomness was tested between the two ordination results with “protest” (vegan); this was visualized with the “procrustes” function (vegan). The last three analyses mentioned use permutations; to increase their accuracy total permutations were increased to 9,999.

### Data Availability Statement

We deposited all 454 sequence data from^16^ in NCBI’s Short Read Archive under BioProject ID PRJNA310230 and all Illumina data were deposited under BioProject ID PRJNA389803.

## Electronic supplementary material

Supplementary Information
