# Supplementary material for: Large-scale differences in microbial biodiversity discovery between 16S amplicon and shotgun sequencing
Source: Sci Rep. 2017 Jul 31;7:6589. doi: 10.1038/s41598-017-06665-3 (PMC5537354; doi:10.1038/s41598-017-06665-3)
Supplement: Supplementary file 1 — Supplementary Information [file 41598_2017_6665_MOESM1_ESM.pdf]

Large-scale differences in microbial biodiversity discovery between 16S amplicon and shotgun sequencing

Michael Tessler [1, 2], Johannes S. Neumann [3], Ebrahim Afshinnekoo [4, 5, 6], Michael Pineda [4, 5], Rebecca Hersch [1], Luiz Felipe M. Velho [7,8], Bianca T. Segovia [7], Fabio A. Lansac-Toha [7], Michael Lemke [9], Rob DeSalle [1], Christopher E. Mason [4, 5, 10] & Mercer R. Brugler [1, 11]

[1]Sackler Institute for Comparative Genomics, American Museum of Natural History, New York, NY 10024, USA. [2]Richard Gilder Graduate School, American Museum of Natural History, New York, NY 10024, USA. [3]Stiftung Tierärztliche Hochschule Hannover, ITZ - Ecology and Evolution, Bünteweg 17d, D-30559 Hannover, Germany. [4]Department of Physiology and Biophysics, Weill Cornell Medicine, New York, NY 10021, USA. [5]The HRH Prince Alwaleed Bin Talal Bin Abdulaziz Alsaud Institute for Computational Biomedicine, New York, NY, 10021, USA. [6]School of Medicine, New York Medical College, Valhalla, NY, 10595, USA. [7]Universidade Estadual de Maringá, Nupelia / PEA, Maringá, Paraná, Brazil. [8]Unicesumar / Instituto Cesumar de Ciência, Tecnologia e Inovação (ICETI), Maringá, Paraná, Brazil. [9]Biology Department, University of Illinois Springfield, Springfield, IL 62703, USA. [10]The Feil Family Brain and Mind Research Institute (BMRI), New York, NY, 10021, USA. [11]Biological Sciences Department, NYC College of Technology, City University of New York, Brooklyn, NY 11201, USA. Correspondence and requests for materials should be addressed to M.T. (email: mtessler@amnh.org), C.E.M. (email: chm2042@med.cornell.edu), or M.R.B. (email: mbrugler@citytech.cuny.edu).

Supplementary Figure 1. Bar plot of finished whole genome sequences for Bacteria and Archaea available from NIH (https://www.ncbi.nlm.nih.gov/genome/browse/ [December 28, 2016]) summarized by phylum. The number of genomes in the three phyla on the right is given above the bar. Purple bars indicate phyla that are present in both the amplicon and shotgun datasets. Green indicates phyla that are present only from shotgun data, red indicates phyla that are present only from amplicon data, and blue are any phyla not found with either method.

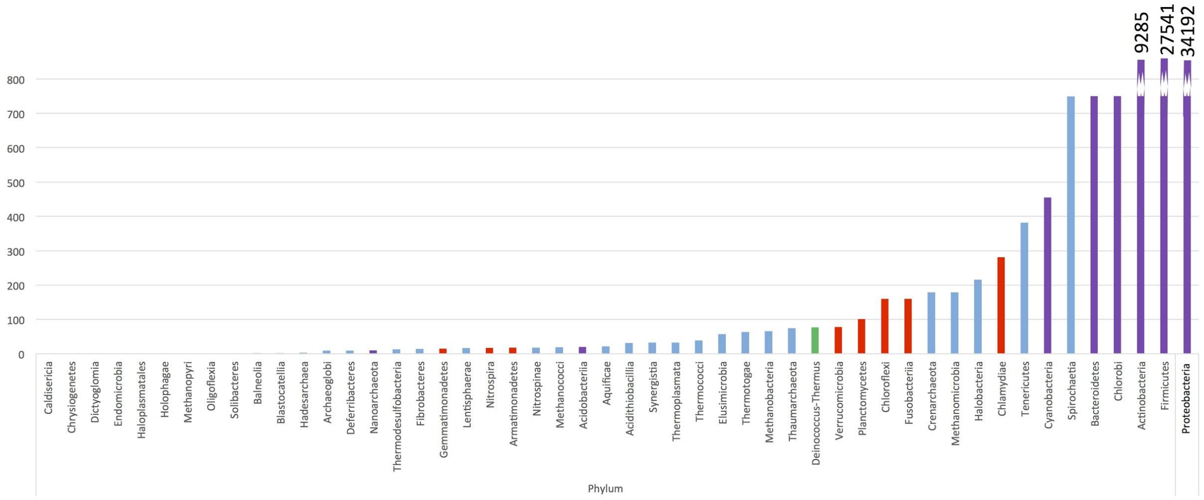

Supplementary Table 1. Correspondence between aquatic environmental variables and NMDS ordinations (Illumina shotgun and 454 amplicon sequencing at the phylum and family levels), as found with vector fitting (envfit), and PERMANOVA*. Significant *p*-values are bolded. First columns summarize significant results; later columns go into detail on r^2 and p values (significant values bolded).

	Phylum		Family		Phylum				Family			
					Shotgun - Illumina		Amplicon - 454		Shotgun - Illumina		Amplicon - 454	
	Shotgun Illumina	Amplicon 454	Shotgun Illumina	Amplicon 454	r^2	p	r^2	p	r^2	p	r^2	p
Chlorophyll (Clorof)	x		x		0.319	**0.001**	0.043	0.392	0.219	**0.006**	0.033	0.489
Conductivity					0.022	0.623	0.002	0.963	0.010	0.811	0.027	0.562
Dissolved oxygen (DO)	x		x	x	0.273	**0.002**	0.121	0.068	0.250	**0.004**	0.257	**0.002**
Dissolved oxygen saturated (DOsat)	x		x	x	0.294	**0.001**	0.093	0.126	0.218	**0.006**	0.190	**0.009**
Depth	x	x			0.257	**0.004**	0.141	**0.039**	0.043	0.407	0.055	0.311
Dissolved inorganic nitrogen (DIN)	x	x			0.326	< **0.001**	0.154	**0.026**	0.037	0.447	0.100	0.114
NH_4	x		x	x	0.298	**0.002**	0.078	0.187	0.059	0.284	0.034	0.482
NO_3	x				0.145	**0.045**	0.160	**0.026**	0.058	0.282	0.106	0.100
pH	x	x			0.075	0.197	0.059	0.283	0.236	**0.003**	0.197	**0.009**
PO_4			x	x	0.004	0.926	0.023	0.617	0.004	0.924	0.020	0.653
Temperature (Tempar)					0.027	0.568	0.108	0.094	0.138	**0.044**	0.121	0.068
Temperature H_2O (TempH2O)			x		0.027	0.567	0.090	0.138	0.163	**0.023**	0.123	0.068
Total nitrogen (TN)			x		0.262	**0.003**	0.081	0.182	0.209	**0.007**	0.035	0.463
Total phosphate (TP)	x		x		0.310	< **0.001**	0.012	0.766	0.214	**0.006**	0.020	0.641
Turbidity (Turb)	x		x		0.223	**0.007**	0.032	0.508	0.080	0.178	0.013	0.756
Z. euphotic (Zeu)	x				0.211	**0.007**	0.147	**0.034**	0.121	0.068	0.113	0.080
Z. max. (Zmax)	x	x			0.257	**0.004**	0.141	**0.039**	0.043	0.407	0.055	0.311
Floodplain*	x	x			0.238	**0.003**	0.092	0.110	0.158	**0.003**	0.111	**0.006**
Total significant	13	5	9	4		13		5		9		4

**Supplementary Table 2.** Total number of taxa found and taxon richness estimates at the phylum and family levels for amplicon and shotgun data (S.E. = standard errors).						
**Rank**	**Method**	**Taxa found**	**Chao**	**Jackknife 1**	**Jackknife 2**	**Bootstrap**
Phylum	Amplicon	20	20.0	20.0	20.0	20.0
	S.E.		0.0	0.0		0.0
	Shotgun	9	14.9	12.9	16.8	10.5
	S.E.		7.0	2.4		1.2
Family	Amplicon	56	56.0	56.0	56.0	56.0
	S.E.		0	0		0.1
	Shotgun	41	54.2	49.8	55.6	44.9
	S.E.		12.2	3.3		1.7

**Supplementary Table 3.** Sample information for the lakes reviewed in our analyses.		
**Locale**	**River**	**Date**
Cadete	Amazon	Oct. 2011
Calado	Amazon	Oct. 2011
Castanho	Amazon	Oct. 2011
Comprido	Amazon	Oct. 2011
Fuxico	Amazon	Oct. 2011
Grande I	Amazon	Oct. 2011
Grande II	Amazon	Oct. 2011
Grande II	Amazon	May. 2012
Lagoa V	Amazon	Oct. 2011
Monte Cristo	Amazon	Oct. 2011
Piranha	Amazon	Oct. 2011
Poço Curuçá	Amazon	Oct. 2011
Poraquequara I	Amazon	Oct. 2011
Poraquequara II	Amazon	Oct. 2011
Poraquequara III	Amazon	Oct. 2011
Rio Amazonas	Amazon	Oct. 2011
Rio Negro	Amazon	Oct. 2011
Rio Solomon	Amazon	Oct. 2011
Tatuí	Amazon	Oct. 2011
Tatuí	Amazon	May. 2012
Crixás I	Araguaia	Nov. 2011
Crixás II	Araguaia	Nov. 2011
Crixás III	Araguaia	Nov. 2011
Crixás IV	Araguaia	Nov. 2011
Goiaba	Araguaia	Mar. 2012
Goiaba	Araguaia	Nov. 2011
Luís Alves II	Araguaia	Nov. 2011
Montaria III	Araguaia	Nov. 2011
Albuquerque	Pantanal	Mar. 2012
Cáceres	Pantanal	Mar. 2012

Figueira	Pantanal	Mar. 2012
Ilha Grande	Pantanal	Mar. 2012
Miranda I	Pantanal	Mar. 2012
Paraguai River	Pantanal	Mar. 2012
Piúva	Pantanal	Mar. 2012
Tuiuiú	Pantanal	Mar. 2012
Aurélio	Paraná	Sept. 2011
Boca do Ipoitã	Paraná	Sept. 2011
Fechada	Paraná	Sept. 2011
Gavião	Paraná	Sept. 2011
Guaraná	Paraná	Sept. 2011
M Luiza	Paraná	Sept. 2011
Onça	Paraná	Sept. 2011
P Garças	Paraná	Sept. 2011
Patos	Paraná	Feb. 2012
Peroba	Paraná	Sept. 2011
Porcos	Paraná	Sept. 2011
Sumida	Paraná	Sept. 2011
Ventura	Paraná	Sept. 2011
